# Supplementary material for: Characterization of the Antiproliferative Activity of Sargassum muticum Low and High Molecular Weight Polysaccharide Fractions
Source: Mar Drugs. 2023 Dec 27;22(1):16. doi: 10.3390/md22010016 (PMC10817663; doi:10.3390/md22010016)
Supplement: Supplementary file 1 [file marinedrugs-22-00016-s001.zip › marinedrugs-2746736-supplementary.pdf]

Supplementary Material

Absence of microorganism contamination in the S. muticum fractions

An aliquot of the samples was seeded in LB agar LENNOX (Condalab, Spain) plates and incubated at 37 °C for 24 – 72 hours to observe any potential bacterial or fungal growth. As negative and positive controls, an aliquot of endotoxin-free and contaminated water, respectively, were used.

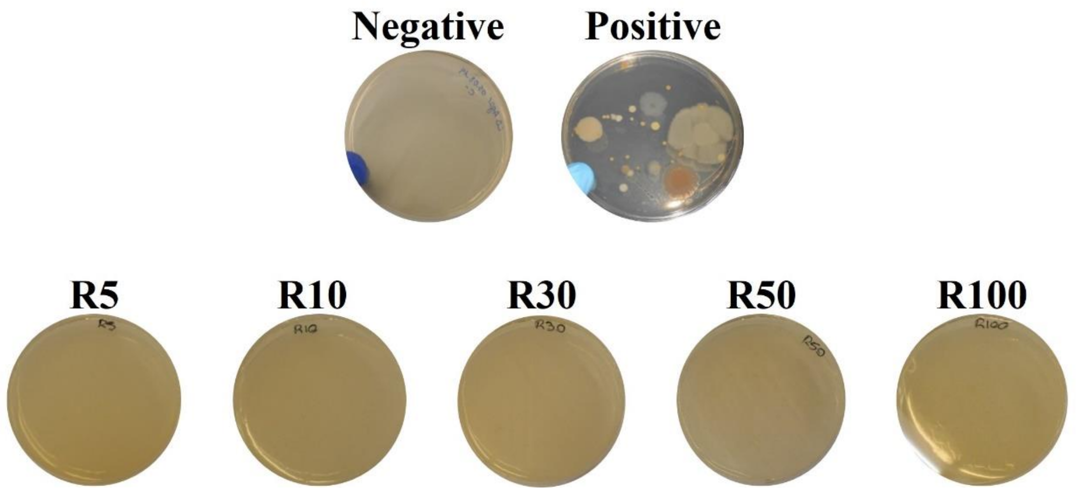

Figure S1. *Characterization of the potential bacterial growth in LB agar plates of an aliquot of R100 (>100 kDa), R50 (50-100 kDa), R30 (30-50 kDa), R10 (10-30 kDa), R5 (5-10 kDa). The plates show the absence of colonies after 72 hours of incubation with the fractions and the negative control, while in the positive control several colonies are clearly observed.*

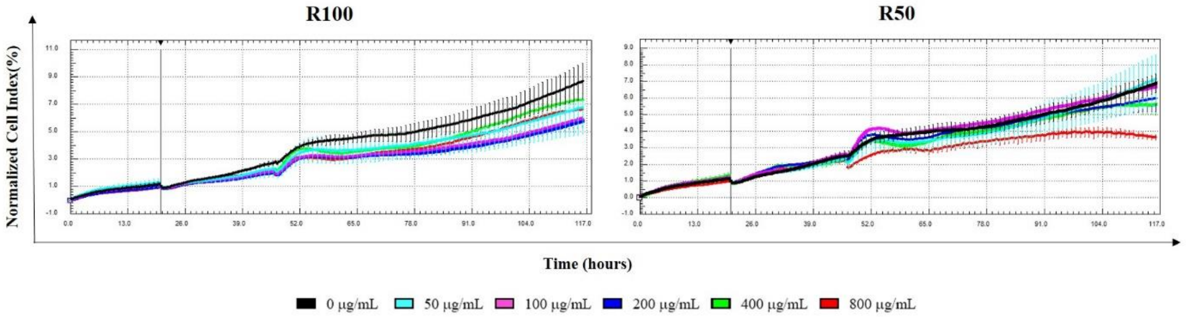

Figure S2. *Kinetics of the normalized cell index of MCF-7 cells after incubation with different concentrations of R50 and R100. The vertical black line represents the time where the extracts were added to the cells.*

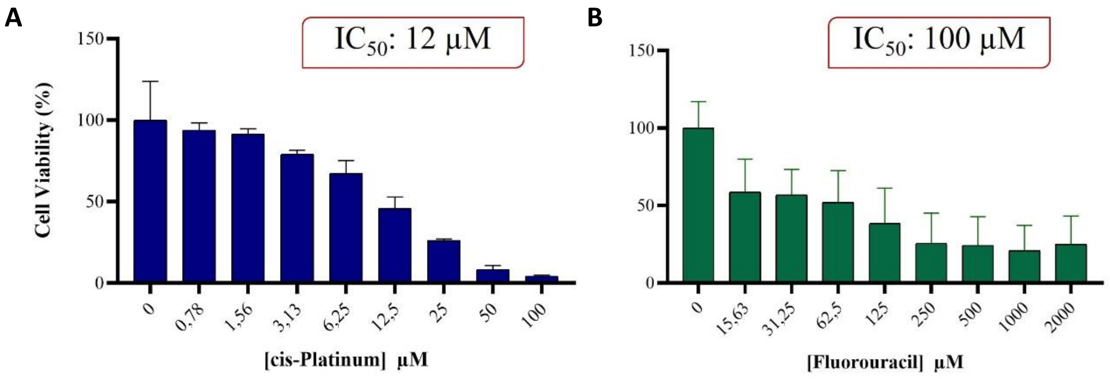

Figure S3. *Percentage of cell viability of MCF-7 cells after 72 hours of incubation with different concentrations of* ***A****) cis-Platinum (cis-Pt) and* ***B****) 5-fluorouracil (5-FU). The IC_{50} was calculated for both compounds and is shown in the graphics.*
